# Supplementary material for: FERN – a Java framework for stochastic simulation and evaluation of reaction networks
Source: BMC Bioinformatics. 2008 Aug 29;9:356. doi: 10.1186/1471-2105-9-356 (PMC2553347; doi:10.1186/1471-2105-9-356)
Supplement: Additional file 1 — FERN distribution, Version 1.3. This archive contains the FERN source code and binaries as well as documentation and example models in FernML and SBML. [file 1471-2105-9-356-S1.zip › fern/doc/javadoc/fern/network/package-frame.html]

fern.network


fern.network

Interfaces 
  
*AnnotationManager*
  
*ComplexDependenciesPropensityCalculator*
  
*Network*
  
*PropensityCalculator*

Classes 
  
AbstractKineticConstantPropensityCalculator
  
AbstractNetworkImpl
  
AmountManager
  
AnnotationManagerImpl
  
ArrayKineticConstantPropensityCalculator
  
NetworkLoader

Exceptions 
  
FeatureNotSupportedException
